# Changes in HIV transmission profile in Arad county

**DOI:** 10.1186/1471-2334-14-S4-P9

**Published:** 2014-05-29

**Authors:** Dana Negru, Teodora Olaru, Laura Nicolescu, Mirandolina Prişcă

**Affiliations:** 1 Public Health Department, Arad, Romania; 2 Arad County Emergency Hospital, Arad, Romania

## 

Initially HIV infection in Arad County was impacting infant population. Later most of the new cases were adults, but even so trends in recent years are moving in the same way for adults and children, the latter being infected through vertical transmission.

The hypothesis was to determine the relationship between age of cases at diagnosis moment and route of transmission in more than two decades from 1990 till now. We have used data from the medical records of HIV-AIDS department of Arad County Emergency Hospital, from 1990 to 2013 statistical processing with SPSS.14.0 for Windows, MedCalc and Epi Info Analysis.

Of the 437 patients, children and young people 1-19 years were 68%, gender ratio M/F being 1.31. Parenteral route of transmission covered 47%, sexual 22%, vertical 2%, and unknown for 29% (statistically significant relationship between age at diagnosis and transmission path p=0.000). Only 149 patients are monitored /34%, 174/40% are out of records and 114/26% died. There are many associated infections like HBV, HCV, TB, CMV, ITS, total 166/38% among total of 437 cases. Relative risk for parenteral transmission was 2.34 in the first decade compared to the second one and reached up to 7.7 for sexual transmission route in the second decade, compared to the first decade.

Pediatric HIV infection versus adult HIV infection rate is changing from 5.53 in the first decade to 0.35 in the second one, risks of sexually transmitted infection becoming dominant for the second decade.

